# Porous Silk Scaffolds for Delivery of Growth Factors and Stem Cells to Enhance Bone Regeneration

**DOI:** 10.1371/journal.pone.0102371

**Published:** 2014-07-22

**Authors:** Wenjie Zhang, Chao Zhu, Dongxia Ye, Ling Xu, Xiaochen Zhang, Qianju Wu, Xiuli Zhang, David L. Kaplan, Xinquan Jiang

**Affiliations:** 1 Department of Prosthodontics, Ninth People's Hospital affiliated to Shanghai Jiao Tong University, School of Medicine, Shanghai, China; 2 Oral Bioengineering and regenerative medicine Lab, Shanghai Research Institute of Stomatology, Ninth People's Hospital Affiliated to Shanghai Jiao Tong University, School of Medicine, Shanghai Key Laboratory of Stomatology, Shanghai, China; 3 Department of Oral and Stomatology, Linyi People's Hospital, Linyi, China; 4 Department of Oral and Maxillofacial Surgery, Ninth People's Hospital, Shanghai JiaoTong University, School of Medicine, Shanghai, China; 5 Department of Biomedical Engineering, School of Engineering, Tufts University, Medford, Massachusetts, United States of America; National University of Ireland, Galway, Ireland

## Abstract

Stem cell-based tissue engineering shows promise for bone regeneration and requires artificial microenvironments to enhance the survival, proliferation and differentiation of the seeded cells. Silk fibroin, as a natural protein polymer, has unique properties for tissue regeneration. The present study aimed to evaluate the influence of porous silk scaffolds on rat bone marrow stem cells (BMSCs) by lenti-GFP tracking both *in vitro* and *in vivo* in cranial bone defects. The number of cells seeded within silk scaffolds in rat cranial bone defects increased from 2 days to 2 weeks after implantation, followed by a decrease at eight weeks. Importantly, the implanted cells survived for 8 weeks *in vivo* and some of the cells might differentiate into endothelial cells and osteoblasts induced by the presence of VEGF and BMP-2 in the scaffolds to promote angiogenesis and osteogenesis. The results demonstrate that porous silk scaffolds provide a suitable niche to maintain long survival and function of the implanted cells for bone regeneration.

## Introduction

Tissue-engineered bone is a relatively new strategy to treat massive bone defects, instead of the use of autologous bone grafts which present drawbacks [1]–[2]. The development of stem cells as a cell-based strategy has also been approved as a promising approach for bone regeneration [3]–[4]. However, a major obstacle to this approach is the survival of transplanted seeded cells [5]–[7]. The long-term survival of seeded cells after transplantation along with biomaterial scaffolds is a prerequisite for the cells to promote tissue regeneration by directly participating in the process or by secreting key growth factors. Therefore, the survival time and fate of the seeded cells *in vivo* plays an important role in influencing the effectiveness of tissue regeneration.

Stem cell fate *in vivo* is controlled by many factors including matrix chemistry and morphology, soluble factors, ions, mechanical forces and other features of the physiological microenvironment, all of which constitute the stem cell niche [8]–[9]. For *in vitro* tissue engineering, synthetic scaffolds serve as the carrier and the living microenvironment for the transplanted stem cells [1], [10]. In order to ensure that the transplanted cells directly participate in tissue regeneration, it is critical to mimic the stem cell niche [8], [11]–[12]. In addition, for bone tissue engineering scaffolds, essential characteristics, such as a highly porous structure, mechanical properties, biocompatibility, slow degradation and suitable surface chemistry are key [13]. With all of these requirements taken into consideration, porous silk scaffolds offer very useful features to meet these needs as a carrier for stem cells in bone tissue engineering.

Silk is biocompatible with low inflammatory and immunogenic responses and has been approved by the FDA for some medical devices [14]. Moreover, silk materials exhibit excellent strength and toughness to meet the requirements for scaffolds for bone tissue engineering [15]. The combination of silk matrices with growth factors also can be employed for bone regeneration [16]–[18]. More importantly, silk-based biomaterials can be tailored for diverse applications [19]; including morphological changes, structural control, and a range of material formats can be prepared such as sponges, hydrogels, fibers, films and other forms [19]. Bio-functional modification of silk materials, changes in elasticity, control of surface roughness [20], biomimetic coatings [21], and collagen incorporation [22] to direct stem cell behavior have all been explored. In total, silk is a useful material for artificial stem cell microenvironment fabrication to deliver seeded cells for bone regeneration, with porous silk scaffolds to facilitate cell survival, proliferation and migration *in vitro* [23]. However, there has been little direct evidence for the fate of stem cells transplanted with silk scaffolds *in vivo*. Thus, the goal of the present study was to address this question by tracking the seeded cells *in vivo* during bone regeneration.

In the present study, the objective was to track stem cell survival *in vivo* to determine if the *in vitro* survival and functions of these cells was valid *in vivo* as a key step toward future clinical translation. CD90+ and CD105+ bone marrow stem cells (BMSCs) from rat femurs were isolated and cultured. Porous silk scaffolds with pore sizes 400–500 µm were used to carry the stem cells for repair of rat critical-sized calvarial defects. The survival of the cells with silk scaffolds *in vivo* was monitored by GFP-labeling for 8 weeks. Furthermore, in order to evaluate biological activity, the differentiation of the implanted cells was studied in combination with angiogenic and osteogenic growth factors.

## Materials and Methods

### Ethics Statement

The Ethics Committee for Animal Research at the Ninth People's Hospital affiliated to Shanghai Jiao Tong University approved all the experimental protocols involving the use of rats.

### Animals

Thirty-three 12-week-old male Fischer 344 rats, weighing about 280 g, were obtained from the Ninth People's Hospital Animal Center (Shanghai, China) for the cranial defect repair experiment, which is a common model to evaluate the *in vivo* bone-forming capacity of tissue-engineered complex. Twelve 4-week-old male Fischer 344 rats, weighing about 70 g, were also obtained from the Ninth People's Hospital Animal Center and used for BMSCs isolation and culture.

### Silk scaffolds

The disk-shaped porous silk fibroin scaffolds with pore sizes 400–500 µm (5 mm in diameter and 2 mm in thick) were fabricated according to our previously published procedures [24]. Recombinant VEGF and BMP-2 proteins used to modify the silk scaffolds were kindly provided by Wyeth [16].

### BMSCs culture

BMSCs were isolated and cultured from 4-week-old rats femurs according to our previously published procedures and totally twelve rats were used to obtain sufficient cells for both *in vitro* and *in vivo* experiments [25]. Firstly, the rats were euthanized with an overdose of pentobarbital by intraperitoneal injection. After separating the femurs and removing remaining surface muscle, both ends of the femur were cut off at the epiphysis and the marrow was quickly rinsed out with DMEM (Gibco BRL, USA) with 10% fetal bovine serum (FBS) (Hyclone, USA).Then the isolated cells were incubated at 37°C in an atmosphere of 5% CO_2_ in DMEM supplemented with 10% FBS, 100 U/ml streptomycin and 100 U/ml penicillin, and 72 hours later, and non-adherent cells were rinsed away using PBS several times. After 7 days the primary cells were fixed in 4% paraformaldehyde and stained with 0.1% toluidine blue to observe cell colonies.

### BMSCs identification

When cells reached about 90% confluence, BMSCs were subcultured into new dishes at a density of 1.0×10^5^ cells/ml using trypsin/EDTA (0.25% w/v trypsin, 0.02% EDTA). For flow cytometry [25], 1.0×10^6^ cells were collected in PBS and sequentially incubated with CD105-PE solution (eBioscience, USA) and CD90-FITC (Invitrogen, USA) for 30 min at 37°C in the dark. Then the samples were assayed on a FACS Calibur flow cytometer (BD). Multilineage differentiation potential of rat BMSCs was further verified by incubation in chondrogenic, adipogenic and osteogenic media (Cyagen, China) as previously reported [26]. For chondrogenic differentiation, 2.5×10^5^ cells were collected into 15 ml polypropylene culture tubes and centrifuged at 150 g for 5 minutes to form cell pellets, and then incubated in the chondrogenic differentiation medium for 21 days. Finally, the cell pellets were prepared into paraffin sections and stained with Alcian Blue. For adipogenic differentiation, cells were fixed and stained using Oil Red O after 21 days induction. ALP staining and calcium nodule formation were used to assess osteogenic differentiation. After 14 days of induction in osteogenic medium, cells were fixed and stained for ALP (Beyotime, China). For calcium nodule formation assay, cells were initially cultured in osteogenic medium for 14 days, and then incubated in osteogenic media with 20 mg/L calcein for another 7 days before observing under a fluorescence microscope.

### Incubating BMSCs with silk scaffolds *in vitro*

Rat BMSCs were transfected with Lenti-EGFP as we have previously described [27]. The cells were infected with Lenti-EGFP at a multiplicity of infection (MOI) of 12 in the presence of 8 µg/ml polybrene. GPF positive (GFP+) cells were further selected by adding 500 µg/ml G418 to the culture medium. Before seeding the cells, porous silk scaffolds were pre-immersed in DMEM with 10% FBS for 24 hours. Then GFP+ cells were collected and resuspended to a concentration of 2×10^7^ cells/ml. A total of 20 µl cell suspension was seeded on each piece of scaffold in 24-well plates. Two hours later, 1 ml of complete medium was added to each well (n = 6). Non-adherent cells were removed by changing medium the next day. The survival of the GFP+ cells on the scaffolds was observed by fluorescence microscopy at 2 days, 2 weeks and 8 weeks, respectively.

### Incubating BMSCs with silk scaffolds *in vivo*

**Figure 1:**
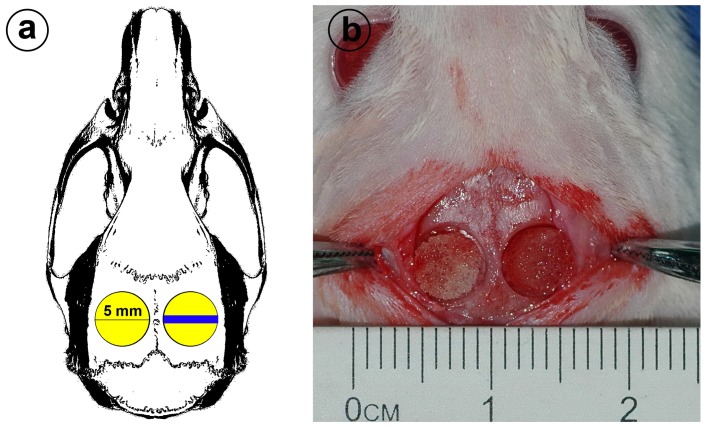
Animal model. (a) Schematic of rat cranial bone defects. Two 5 mm diameter critical size defects were made on the skull. The blue area shows the location where the tissue was taken for immunohistochemistry. (b) Rat cranial bone defects with silk scaffold-grafted.

A rat calvarial defect model was used to evaluate the survival of BMSCs within the silk scaffolds *in vivo*. According to our previously published procedures [27] (Figure 1), bilateral critically-sized full thickness defects (5 mm in diameter) were made in both sides of the animal skulls. Nine rats were anesthetized by an intraperitoneal injection of pentobarbital (Nembutal 3.5 mg/100 g). Totally eighteen defects were created and then filled with the silk scaffolds seeded with 20 µl GFP+ BMSCs suspension at a concentration of 2×10^7^ cells/ml. At 2 days, 2 weeks and 8 weeks after the operation, three rats were sacrificed with an overdose of pentobarbital and the implanted constructs were explanted, respectively (n = 6 for each time point). To observe GFP+ cells in the scaffolds, the samples were embedded in Tissue-tek OCT compound, and 5 µm thick sections were cut using a cryomicrotome. Before observed under Confocal Laser Scanning Microscopy (CLSM, Leica, Germany), all sections were counterstained with DAPI (Invitrogen). In the right circular defect (Figure 1a) the blue area represents the location selected for histological observation.

### BMSCs differentiation induced by VEGF and BMP-2 *in vivo*

The calvarial defect model described above was used to evaluate the differentiation capacity of the BMSCs upon induction with VEGF and BMP-2. For growth factor loading, each scaffold carried 2 µg VEGF and 3 µg BMP-2, and the scaffolds were stored at −80°C before seeding cells. For BMSCs seeding, a 20 µl cell suspension at a concentration of 2×10^7^ cells/ml was added on each porous scaffold. A total of 4 study groups were used: (a) silk scaffolds alone (Silk group); (b) silk scaffolds seeded with BMSCs (S+cells group); (c) silk scaffolds loaded with VEGF and BMP-2 (S+V/B group); (d) silk scaffolds loaded with VEGF and BMP-2 and the BMSCs (S+V/B+cells group). For evaluation of the osteogenic potential, twelve rats were anesthetized by an intraperitoneal injection of pentobarbital, calvarial defects were created and silk scaffolds of the different study groups were placed in the defects randomly. After 8 weeks, the rats were sacrificed with an overdose of pentobarbital and specimens harvested and fixed in 10% buffered formaldehyde solution. The area of newly formed bone within defect region was captured by scanning with an x-ray tube with potential of 80 kV, a tube current of 0.45 mA and 15 µm voxel resolution using a desktop Micro-CT system (μCT-80, Scanco Medical, Switzerland) as we have previously described [28]. For assessment of vascularization, another six rats implanted with scaffolds of the four groups were perfused with Microfil (Flow Tech, USA) as we have reported [29]–[30]. The rats were anesthetized with an intraperitoneal injection of pentobarbital, and the left ventricle was exposed and penetrated with an angiocatheter after the descending aorta was clamped. And then, 20 ml of Microfil was perfused at 2 ml/min following perfusion with saline. Finally, the rats were set at 4°C for 2 hours before collecting the specimens. After fixing in 10% buffered formaldehyde solution, blue colored Microfil labeled rat skulls were dehydrated using a series of ascending concentrations of alcohols from 25% to 100% and then placed into dimethylbenzene to transparentize the tissue. Digital pictures were captured with a Nikon digital camera and the areas of newly formed blood vessels were quantified using ImageJ software [30].

### Tracking BMSCs by immunohistochemical assay

To detect whether the implanted BMSCs differentiated into blood-forming cells and bone-forming cells induced by the presence of VEGF and BMP-2, an additional six rats were implanted with GFP+ BMSCs under general anesthesia with an intraperitoneal injection of pentobarbital. At 8 weeks after the operation, the rats were sacrificed with an overdose of pentobarbital. The samples were extracted and fixed in 4% paraformaldehyde for 1 day, and then decalcified in 15% EDTA for 2–3 weeks. After embedding in paraffin, a series of 5 mm sections were cut along the coronal cross using a microtome. For immunohistochemical detection, the sections were deparaffinized and hydrated through xylene and graded alcohols, and incubated with primary antibodies against GFP (1∶400 dilution; Abcam, USA). HRP-labeled secondary antibody and DAB substrate were sequentially added to detect the GFP+ cells in the specimens. Cell nuclei were slightly counterstained with hematoxylin.

### Statistical analysis

All data are expressed as the mean ± SD. Statistically significant differences (*p*<0.05 or *p*<0.01) among the various groups were measured with One-way ANOVA and Student-Newman-Keuls (SNK) using a SAS 8.2 statistical software package.

## Results

### Identification of rat BMSCs

**Figure 2:**
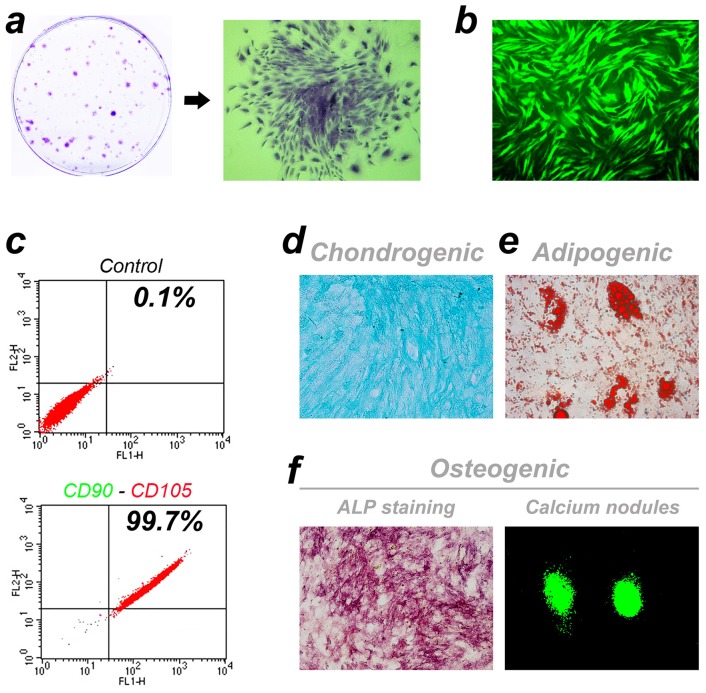
Cell culture and identification. (a) Cell colonies of different sizes observed after 7 days of primary culture. (b) Spindle shaped BMSCs of passage 2 labeled with GFP. (c) CD90 and CD105 co-expression by cells detected by FACS. (d-f) Multi-directional differentiation potential assay of rat BMSCs.

For the BMSCs, many colonies of different sizes were observed after 7 days of primary culture and it was difficult to find discrete single cells under the microscope (Figure 2a). After subculture, the GFP labeled BMSCs showed spindle shapes and the presence of vortex-like growth (Figure 2b). Based on the cytometry results, up to 99.71% of the cultured cells positively expressed CD90 and CD105 simultaneously (Figure 2c). We also tested the multi-directional differentiation potential of rat BMSCs. After 3 weeks of induction, the CD90+ and CD105+ cells developed into Alcian Blue positive chondrocytes (Figure 2d), Oil Red O positive adipocytes (Figure 2e) and osteoblasts with ALP positive staining and calcium nodule-formation (Figure 2f).

### Fate of rat BMSCs with silk scaffolds *in vitro*

**Figure 3:**
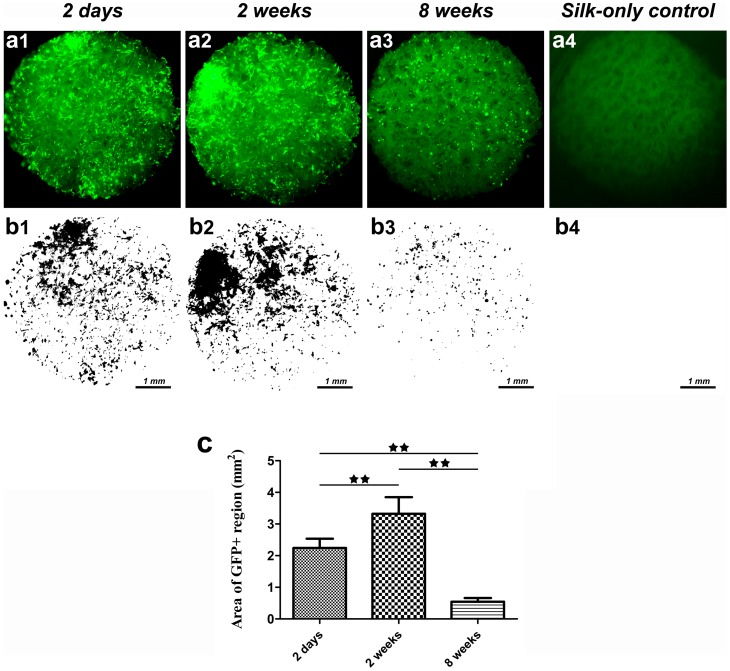
The fate of rat BMSCs cultured with silk scaffolds *in vitro*. (a) Sequential observation of GFP labelled cells in the scaffolds by fluorescence microscopy. (b) Black-white images processed using Image J software. (c) The graph shows the area of GFP positive regions for the different study groups. (★★, represents *p*<0.01).

Rat BMSCs labeled with GFP were seeded in the porous silk scaffolds and incubated in DMEM *in vitro*. After 2 days, 2 weeks and 8 weeks of culture, changes of GFP+ cells in the scaffold were recorded using a fluorescence microscope. The GFP+ cells pervaded the whole scaffold at 2 days (Figure 3a). By comparison, more cells were observed at 2 weeks and we concluded that the silk scaffold was favorable for promoting the survival and proliferation of rat BMSCs. By the eighth week, cell numbers decreased, leaving scattered GFP+ cells in the scaffolds. To quantify the amount of GFP+ cells at different time points, green fluorescent images were transformed into black-and-white pictures (Figure 3b) and the slightly green glow from silk autofluorescence was eliminated by adjusting the threshold using ImageJ software. As shown in Figure 3c, the area of the GFP+ regions within the whole scaffold at week 2 was significantly higher than that at day 2 (*p*<0.01), and the area at week 8 was only 0.54±0.16 mm^2^, which is less compared to the two earlier groups (*p*<0.01).

### Survival of implanted cells with silk scaffolds in rat cranial defects

**Figure 4:**
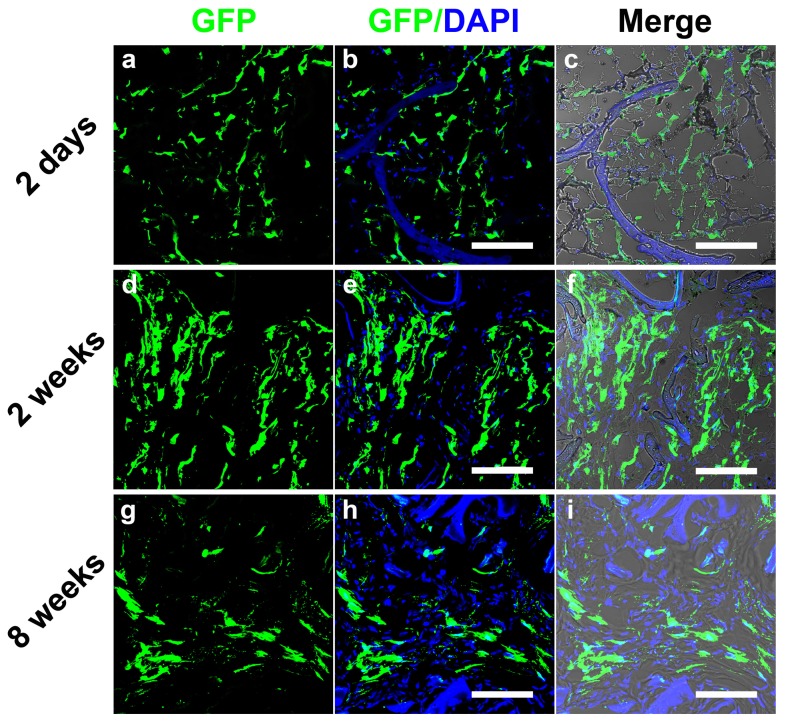
Sequential observation of GFP labelled cells in the silk scaffolds in rat cranial bone defects for 8 weeks. 'Merge' represented the merged images of GFP/DAPI image and transmitted light image. Scale bars are 50 µm.

**Figure 5:**
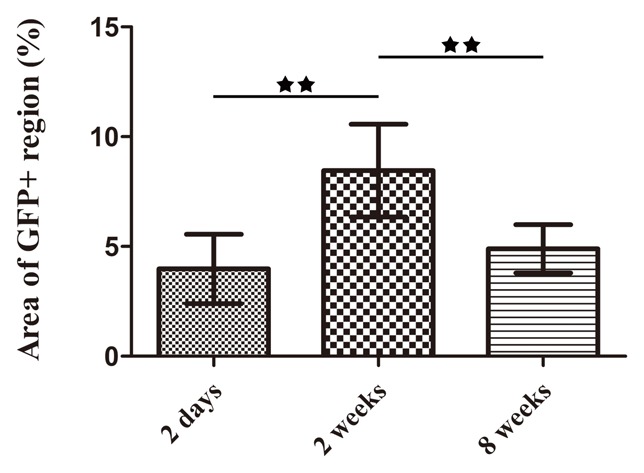
Histomorphometric analysis. The graph shows the area of GFP positive regions for the different study groups. (★★, represents *p*<0.01).

The constructs consisting of GFP+ cells and silk scaffolds were extracted and observed under CLSM at 2 days, 2 weeks and 8 weeks after implantation (Figure 4). At 2 days, many GFP+ cells survived and were observed within the scaffolds. The GFP+ cells adhered to the silk scaffold surfaces, and extensive matrix penetrated into the scaffolds through interconnected pores and might also served as the carrier for the implanted cells (Figure 4c). In contrast, 2 weeks after implantation, the amount of GFP+ cells increased in the scaffold pores (*p*<0.01) (Figure 4f and Figure 5). Over time, the number of GFP+ cells dramatically decreased at 8 weeks when compared to the 2 week group (*p*<0.01). The proportion of GFP+ cells at 8 weeks also decreased when compared to the 2 day group (Figure 4h). However, as shown in Figure 5, there was no obvious difference in the total amount of GFP+ cells between 2 days and 8 weeks, which suggests that the porous silk scaffolds served as cell carriers *in vivo* to facilitate the implanted cells survival for extended time frames.

### Bone formation evaluated by radiographic analysis

**Figure 6:**
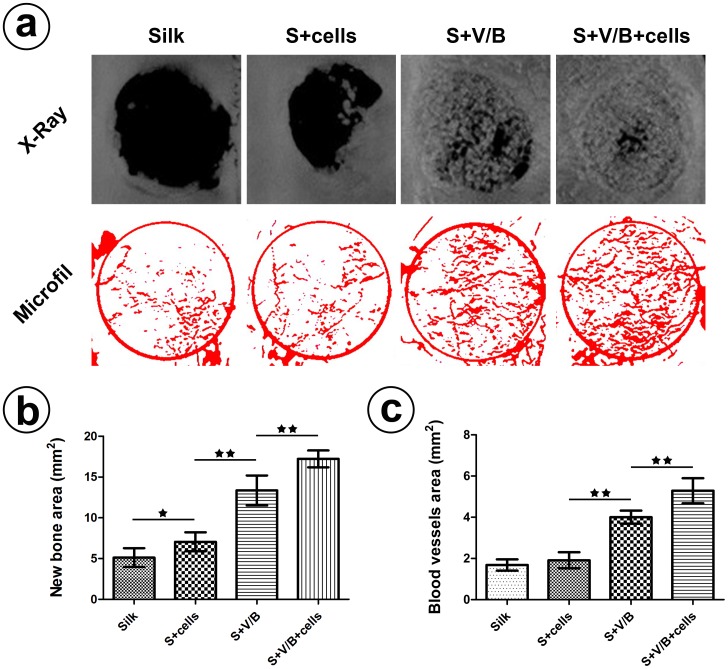
Bone and blood vessels formation assay. (a)Representative X-ray images and images of Microfil labeled blood vessels analyzed using Image J. (b) Analysis of the new bone area in the cranial bone defects. (c) Analysis of the local newly formed blood vessel area in the defects. (★, represents *p*<0.05; ★★, represents *p*<0.01).

To evaluate new bone formation induced by the presence of VEGF and BMP-2, the specimens were collected 8 weeks after implantation and X-ray images were obtained to calculate the area of new bone formation. More new bone was observed in the two groups loaded with VEGF and BMP-2 (Figure 6a), which is in agreement with the Micro-CT observations (Figure S1). The new bone area in group S+V/B and group S+V/B+cells were 13.35±1.85 mm^2^ and 17.23±1.04 mm^2^, respectively. There was a significant difference between the two groups (*p*<0.01) (Figure 6b). In addition, the comparison between group Silk (5.13±1.15 mm^2^) and group S+cells (7.06±1.17 mm^2^) showed a statistically significant difference (*p*<0.05). However, the gap between the Silk group and the S+cells group was less than that between the S+V/B group and S+V/B+cells group. We conclude from the results that the growth factors play an important role in promoting bone formation from stem cells.

### Blood vessel formation displayed by Microfil infusion

At 8 weeks after implantation the rats used for the evaluation of vascularization were sacrificed with an overdose of pentobarbital and perfused with Microfil. After the transparentizing process (Figure S2), blood vessels labeled by blue-colored Microfil were vividly displayed in the defects and the density of blood vessels in group S+V/B and group S+V/B+cells are higher than in the other two groups. Blood vessels were extracted from the original digital images via ImageJ and used to reconstruct the blood vessel area (Figure 6a). There was no significant difference in the extent of vascularization between group Silk and group S+cells according to the statistical results (Figure 6c). However, the area of newly formed blood vessels in the S+V/B+cells group was significantly increased when compared with group S+V/B (*p*<0.05). All of the results demonstrate that the implanted growth factors promoted rat BMSCs to participate in blood vessel formation.

### Cells tracking in rat cranial defects

**Figure 7:**
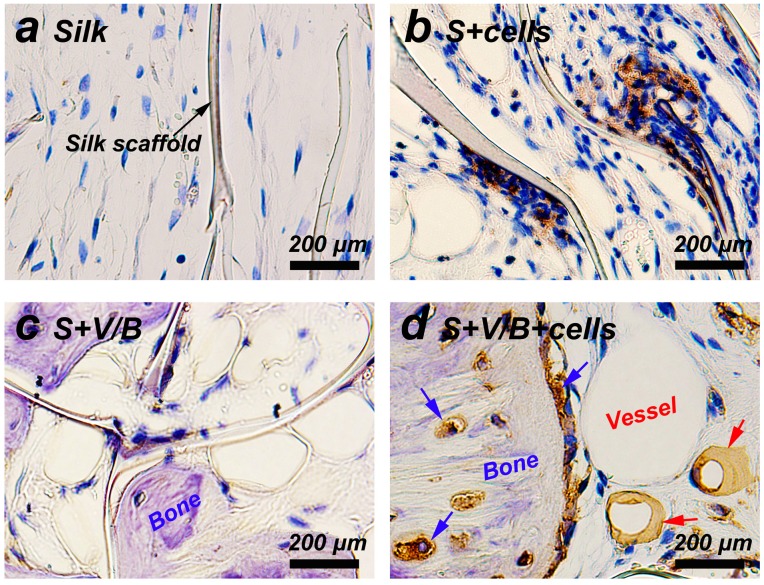
Immunostaining for GFP in each group at 8 weeks post-operation. GFP positive cells stained brownish yellow (b and d). Black arrow shows remnant of silk scaffold. Blue arrows indicate osteoblasts and osteocytes. Red arrows indicate endothelial cells.

The implanted cells were detected using immunohistochemistry for GFP. As shown in Figure 7b and d, many GFP+ cells were still observed in both the S+cells group and S+V/B+cells group at 8 weeks after surgery. More importantly, the implanted GFP positive cells in group S+V/B+cells were detected both in the newly formed blood vessels and bone tissues. It was interesting to note that the seeded cells within silk scaffolds not only survived after 8 weeks *in vivo*, but might also functionally differentiate into osteocytes, osteoblasts and endothelial cells under the influence of BMP-2 and VEGF.

## Discussion

It is generally recognized that stem cells show significant promise in tissue engineering and in promoting tissue regeneration [3], [31]–[32]. In the process of tissue engineering and regeneration scaffolds offer an essential microenvironment for seeded cells to reside in and carry out their functions [33]. Therefore, the exploration of favorable biomaterials for cell survival is important. The current study evaluated the effect of porous silk scaffolds carrying BMSCs for bone regeneration in rat cranial defects. The survival and differentiation of the implanted cells were traced with GFP labeling.

Silk is a natural protein polymer which has been widely used in biomedical applications. Silk exhibits comparable or even more favorable biocompatibility when compared other biomaterials such as collagen and polylactic acid [14]. Previous studies confirmed the advantages of porous silk scaffolds to support cell cultures [23], [34]. The scaffolds with interconnected pores play an important role in promoting cell proliferation and migration, while also maintaining good mass transfer for oxygen and nutrients during tissue formation or regeneration [23]. After initial seeding of rat BMSCs, cells rapidly adhered to and spread on the silk matrices *in vitro*. Sufficient nutrition supply through the interconnect pores promoted cell proliferation at this early stage. In this study, the cells seeded in the porous silk scaffolds grew well both *in-vitro* and *in-vivo* from 2 days to 2 weeks. Although the number of cells decreased *in-vitro* at 8 weeks, the number of cells implanted *in-vivo* with silk scaffolds stayed at a high level. The decrease of cells numbers *in-vitro* may be caused by culture conditions which do not completely mimic the environment of cells *in situ*. Silk is also a mechanically robust biomaterial with predictable long-term degradation characteristics [35]. Topically, the porous silk scaffolds were a suitable carrier for delivering seeded cells for bone tissue engineering by facilitating the survival and maintaining the proliferation of the cells.

BMSCs, with multi-directional differentiation potential, are suitable seeded cells for tissue engineering [36]–[37]. As a relatively accessible source for therapeutic use, BMSCs have been widely adopted in promoting bone regeneration [32], [38]. The number of seeded cells is critical for successful bone regeneration, and the amount of newly formed bone increases with more cells within a suitable range [39]. It is generally assumed that BMSCs secrete cytokines and growth factors or directly differentiate into osteoblasts to participate in bone regeneration [40]. In addition, these cells are able to differentiate into endothelial cells to enhance the formation of new bone [29]. Therefore, it is beneficial for the quality of new bone to prolong the lifespan and enhance the bioactivity of implanted cells *in vivo*. Our present results demonstrate the advantages of porous silk scaffold as a carrier for BMSCs for rat cranial bone defects. A large number of GFP+ cells were still detected at 8 weeks after implantation. To further confirm whether the biological function of the implanted cells was influenced over this time frame of incubation within the silk scaffolds, VEGF and BMP-2 were delivered to direct the differentiation of those cells.

VEGF and BMP-2 have been well documented with the potent capacity to promote angiogenesis and osteogenesis [41]–[44]. They are two important regulators extensively used to induce endothelial and osteogenic differentiation of stem cells [45]–[48]. The combination of VEGF and BMP-2 is the most common growth factor group used in the bone regeneration for their synergistic effects [49]–[50]. Recently, we further confirmed the ability of VEGF and BMP-2 transported by silk hydrogels to enhance vacularized bone regeneration in the elevated sinus cavity [16]. Silk has several unique properties making it a favorable matrix for the incorporation and delivery of growth factors [51]. The porous silk scaffold, used in this study, also has been used for delivering BMP-2 for bone regeneration, and there was still 25% of the initial BMP-2 retained after culturing in the medium for 1 week [17]. In the present study, the combination of VEGF and BMP-2 was incorporated into the silk scaffolds to induce BMSCs differentiation in the rat cranial bone defects. According to both angiogenesis and osteogenesis results, the differences between the S+V/B group and S+V/B+cells group was larger than that between the Silk group and the S+cells group, which indicated that the loaded growth factors enhanced the ability of BMSCs to participate in vascularized bone regeneration. In contrast to previous studies, the seeded CD90+ and CD105+ BMSCs did not undergo osteogenic induction *in vitro*. Using a lenti-GFP labeling strategy, we further detected that the implanted cells in group S+V/B+cells eventually located both in the newly formed blood vessels and bone tissues, which indicated that the implanted cells might differentiate into endothelial cells and osteoblasts induced by VEGF and BMP-2.

Dynamic *in vivo* tracking of stem cells in animal models is essential for optimizing treatments [52]. Various strategies have been developed to label cells, amongst which GFP labelling is a powerful tool to follow the fate of the transplanted cells [53]. While stem cell-based bone tissue engineering has been well-documented in the past decade, only a few studies have reported on real time tracking of cell fate. Polycaprolactone (PCL) scaffolds have served as a BMSC carrier for femur segmental bone defects, and cell numbers decreased significantly at 2 weeks post-implantation [54]. In other studies, a chitosan and glycerophosphate gel carrying BMSCs was used in osteochondral defects, and the cells migrated into the surrounding tissue without actively participating in defect repair [55]. Accordingly, we speculate that different defect regions and specifics of the repair requirements may impact cell fate. More importantly, stem cell carriers, the artificial microenvironment, play a more important role, inasmuch as we also detect that the implanted cells only adhere to the surface of calcium phosphate cements (CPC) scaffold 2 weeks later after implantation in rat cranial bone defects (Figure S3), distinguished from silk scaffolds. The possible reason is that favorable biological characteristics and suitable aperture size of the porous silk scaffold facilitate blood serum proteins adhesion to supply living space for more implanted cells (Figure 4c).

## Conclusion

The present study shows that porous silk scaffolds promoted rat cells survival and proliferation both *in vitro* and *in vivo*. More importantly, the implanted cells survived for long time frames *in vivo*, and also play an important role in promoting angiogenesis and osteogenesis with the stimulation of VEGF and BMP-2. The results provide direct support that porous silk scaffolds can serve as a suitable carrier for seed cells for bone regeneration. The results also indicated the possible differentiation capacity of BMSCs, without osteogenic induction before implantation *in vitro*, into endothelial cells and osteoblasts induced by the presence of VEGF and BMP-2.

## Supporting Information

Figure S1 **Micro-CT analysis of the repaired skull 8 weeks after implantation.** (a) The apical and antapical views of three-dimensional reconstruction image. Bone volume (b) and trabecular number (c) were measured. (★, represents *p*<0.05; ★★, represents *p*<0.01).
(TIF)

Figure S2 **Observation of blue Microfil-perfused blood vessels in the gross specimens.** Scale bars are 1 mm.
(TIF)

Figure S3 **Cell tracking within calcium phosphate cement (CPC) scaffolds.** The red dash lines indicates the CPC scaffold surface.
(TIF)

## References

[1] Langer R, Vacanti JP (1993) Tissue engineering. Science 260: 920–926. 8493529 10.1126/science.8493529

[2] Petite H, Viateau V, Bensaid W, Meunier A, De Pollak C, et al (2000) Tissue-engineered bone regeneration. Nat Biotechnol 18: 959–963. 10973216 10.1038/79449

[3] Bianco P, Robey PG (2001) Stem cells in tissue engineering. Nature 414: 118–121. 11689957 10.1038/35102181

[4] Yamada Y, Ito K, Nakamura S, Ueda M, Nagasaka T (2011) Promising cell-based therapy for bone regeneration using stem cells from deciduous teeth, dental pulp, and bone marrow. Cell Transplant 20: 1003–1013. 21054950 10.3727/096368910X539128

[5] Vogel G (2011) Mending the Youngest Hearts. Science 333: 1088–1089. 21868648 10.1126/science.333.6046.1088

[6] Zimmermann CE, Gierloff M, Hedderich J, Açil Y, Wiltfang J, et al (2011) Survival of transplanted rat bone marrow-derived osteogenic stem cells in vivo. Tissue Eng Part A 17: 1147–1156. 21142699 10.1089/ten.TEA.2009.0577

[7] Zhang ZY, Teoh SH, Chong MS, Lee ES, Tan LG, et al (2010) Neo-vascularization and bone formation mediated by fetal mesenchymal stem cell tissue-engineered bone grafts in critical-size femoral defects. Biomaterials 31: 608–620. 19836073 10.1016/j.biomaterials.2009.09.078

[8] Discher DE, Mooney DJ, Zandstra PW (2009) Growth factors, matrices, and forces combine and control stem cells. Science 324: 1673–1677. 19556500 10.1126/science.1171643 PMC2847855

[9] Wagers AJ (2012) The stem cell niche in regenerative medicine. Cell Stem Cell 10: 362–369. 22482502 10.1016/j.stem.2012.02.018

[10] Vacanti JP, Langer R (1999) Tissue engineering: the design and fabrication of living replacement devices for surgical reconstruction and transplantation. Lancet 354: S32–S34. 10.1016/s0140-6736(99)90247-7 10437854

[11] Colnot C (2011) Cell sources for bone tissue engineering: insights from basic science. Tissue Eng Part B Rev 17: 449–457. 21902612 10.1089/ten.teb.2011.0243 PMC3223018

[12] Kuraitis D, Giordano C, Ruel M, Musarò A, Suuronen EJ (2012) Exploiting extracellular matrix-stem cell interactions: A review of natural materials for therapeutic muscle regeneration. Biomaterials 33: 428–443. 22014942 10.1016/j.biomaterials.2011.09.078

[13] Hutmacher DW (2000) Scaffolds in tissue engineering bone and cartilage. Biomaterials 21: 2529–2543. 11071603 10.1016/s0142-9612(00)00121-6

[14] Altman GH, Diaz F, Jakuba C, Calabro T, Horan RL, et al (2003) Silk-based biomaterials. Biomaterials 24: 401–416. 12423595 10.1016/s0142-9612(02)00353-8

[15] Jin HJ, Kaplan DL (2003) Mechanism of silk processing in insects and spiders. Nature 424: 1057–1061. 12944968 10.1038/nature01809

[16] Zhang W, Wang X, Wang S, Zhao J, Xu L, et al (2011) The use of injectable sonication-induced silk hydrogel for VEGF165 and BMP-2 delivery for elevation of the maxillary sinus floor. Biomaterials 32: 9415–9424. 21889205 10.1016/j.biomaterials.2011.08.047 PMC3384686

[17] Karageorgiou V, Tomkins M, Fajardo R, Meinel L, Snyder B, et al (2006) Porous silk fibroin 3-D scaffolds for delivery of bone morphogenetic protein-2 in vitro and in vivo. J Biomed Mater Res A 78: 324–334. 16637042 10.1002/jbm.a.30728

[18] Li C, Vepari C, Jin HJ, Kim HJ, Kaplan DL (2006) Electrospun silk-BMP-2 scaffolds for bone tissue engineering. Biomaterials 27: 3115–3124. 16458961 10.1016/j.biomaterials.2006.01.022

[19] Omenetto FG, Kaplan DL (2010) New opportunities for an ancient material. Science 329: 528–531. 20671180 10.1126/science.1188936 PMC3136811

[20] Hu X, Park SH, Gil ES, Xia XX, Weiss AS, et al (2011) The influence of elasticity and surface roughness on myogenic and osteogenic-differentiation of cells on silk-elastin biomaterials. Biomaterials 32: 8979–8989. 21872326 10.1016/j.biomaterials.2011.08.037 PMC3206257

[21] Jiang X, Zhao J, Wang S, Sun X, Zhang X, et al (2009) Mandibular repair in rats with premineralized silk scaffolds and BMP-2-modified bMSCs. Biomaterials 30: 4522–4532. 19501905 10.1016/j.biomaterials.2009.05.021 PMC2871698

[22] An B, DesRochers TM, Qin G, Xia X, Thiagarajan G, et al (2013) The influence of specific binding of collagen–silk chimeras to silk biomaterials on hMSC behavior. Biomaterials 34: 402–412. 23088839 10.1016/j.biomaterials.2012.09.085 PMC3490004

[23] Mandal BB, Kundu SC (2009) Cell proliferation and migration in silk fibroin 3D scaffolds. Biomaterials 30: 2956–2965. 19249094 10.1016/j.biomaterials.2009.02.006

[24] Nazarov R, Jin HJ, Kaplan DL (2004) Porous 3-D scaffolds from regenerated silk fibroin. Biomacromolecules 5: 718–726. 15132652 10.1021/bm034327e

[25] Zhang W, Li Z, Liu Y, Ye D, Li J, et al (2012) Biofunctionalization of a titanium surface with a nano-sawtooth structure regulates the behavior of rat bone marrow mesenchymal stem cells. Int J Nanomedicine 7: 4459–4472. 22927760 10.2147/IJN.S33575 PMC3422101

[26] Pittenger MF, Mackay AM, Beck SC, Jaiswal RK, Douglas R, et al (1999) Multilineage potential of adult human mesenchymal stem cells. Science 284: 143–147. 10102814 10.1126/science.284.5411.143

[27] Zhu C, Chang Q, Zou D, Zhang W, Wang S, et al (2011) LvBMP-2 gene-modified BMSCs combined with calcium phosphate cement scaffolds for the repair of calvarial defects in rats. J Mater Sci Mater Med 22: 1965–1973. 21681654 10.1007/s10856-011-4376-6

[28] Zou D, Zhang Z, Ye D, Tang A, Deng L, et al (2011) Repair of critical-sized rat calvarial defects using genetically engineered bone marrow-derived mesenchymal stem cells overexpressing hypoxia-inducible factor-1alpha. Stem Cells 29: 1380–1390. 21774039 10.1002/stem.693

[29] Zou D, Zhang Z, He J, Zhang K, Ye D, et al (2012) Blood vessel formation in the tissue-engineered bone with the constitutively active form of HIF-1α mediated BMSCs. Biomaterials 33: 2097–2108. 22172336 10.1016/j.biomaterials.2011.11.053

[30] Zhang W, Zhu C, Wu Y, Ye D, Wang S, et al (2014) VEGF and BMP-2 promote bone regeneration by facilitating bone marrow stem cell homing and differentiation. Eur Cell Mater 27: 1–12. 10.22203/ecm.v027a01 24425156

[31] Huang GT, Gronthos S, Shi S (2009) Mesenchymal stem cells derived from dental tissues vs. those from other sources: their biology and role in regenerative medicine. J Dent Res 88: 792–806. 19767575 10.1177/0022034509340867 PMC2830488

[32] Zhang W, Zhang X, Wang S, Xu L, Zhang M, et al (2013) Comparison of the Use of Adipose Tissue–Derived and Bone Marrow–Derived Stem Cells for Rapid Bone Regeneration. J Dent Res 92: 1136–1141. 24097853 10.1177/0022034513507581

[33] Shin H (2007) Fabrication methods of an engineered microenvironment for analysis of cell–biomaterial interactions. Biomaterials 28: 126–133. 16945407 10.1016/j.biomaterials.2006.08.007

[34] Meinel L, Fajardo R, Hofmann S, Langer R, Chen J, et al (2005) Silk implants for the healing of critical size bone defects. Bone 37: 688–698. 16140599 10.1016/j.bone.2005.06.010

[35] Wang Y, Rudym DD, Walsh A, Abrahamsen L, Kim HJ, et al (2008) In vivo degradation of three-dimensional silk fibroin scaffolds. Biomaterials 29: 3415–3428. 18502501 10.1016/j.biomaterials.2008.05.002 PMC3206261

[36] Jiang Y, Jahagirdar BN, Reinhardt RL, Schwartz RE, Keene CD, et al (2002) Pluripotency of mesenchymal stem cells derived from adult marrow. Nature 418: 41–49. 12077603 10.1038/nature00870

[37] Pittenger MF, Mackay AM, Beck SC, Jaiswal RK, Douglas R, et al (1999) Multilineage potential of adult human mesenchymal stem cells. Science 284: 143–147. 10102814 10.1126/science.284.5411.143

[38] Mankani MH, Kuznetsov SA, Wolfe RM, Marshall GW, Robey PG (2006) In vivo bone formation by human bone marrow stromal cells: reconstruction of the mouse calvarium and mandible. Stem Cells 24: 2140–2149. 16763200 10.1634/stemcells.2005-0567

[39] Pieri F, Lucarelli E, Corinaldesi G, Aldini NN, Fini M, et al (2010) Dose-dependent effect of adipose-derived adult stem cells on vertical bone regeneration in rabbit calvarium. Biomaterials 31: 3527–3535. 20170950 10.1016/j.biomaterials.2010.01.066

[40] Egusa H, Sonoyama W, Nishimura M, Atsuta I, Akiyama K (2012) Stem cells in dentistry – Part II: Clinical applications. J Prosthodont Res 56: 229–248. 23137671 10.1016/j.jpor.2012.10.001

[41] Street J, Bao M, DeGuzman L, Bunting S, Peale FV, et al (2002) Vascular endothelial growth factor stimulates bone repair by promoting angiogenesis and bone turnover. Proc Natl Acad Sci U S A 99: 9656–9661. 12118119 10.1073/pnas.152324099 PMC124965

[42] Chen D, Zhao M, Mundy GR (2004) Bone morphogenetic proteins. Growth Factors 22: 233–241. 15621726 10.1080/08977190412331279890

[43] Langenfeld EM, Langenfeld J (2004) Bone Morphogenetic Protein-2 Stimulates Angiogenesis in Developing Tumors. Mol Cancer Res 2: 141–149. 15037653

[44] Coultas L, Chawengsaksophak K, Rossant J (2005) Endothelial cells and VEGF in vascular development. Nature 438: 937–945. 16355211 10.1038/nature04479

[45] Behr B, Tang C, Germann G, Longaker MT, Quarto N (2011) Locally Applied Vascular Endothelial Growth Factor A Increases the Osteogenic Healing Capacity of Human Adipose-Derived Stem Cells by Promoting Osteogenic and Endothelial Differentiation. Stem Cells 29: 286–296. 21732486 10.1002/stem.581 PMC3400547

[46] Reyes M, Dudek A, Jahagirdar B, Koodie L, Marker PH, et al (2002) Origin of endothelial progenitors in human postnatal bone marrow. J Clin Invest 109: 337–346. 11827993 10.1172/JCI14327 PMC150857

[47] Jaiswal N, Haynesworth SE, Caplan AI, Bruder SP (1997) Osteogenic differentiation of purified, culture-expanded human mesenchymal stem cells in vitro. J Cell Biochem 64: 295–312. 9027589

[48] Raida M, Heymann A, Günther C, Niederwieser D (2006) Role of bone morphogenetic protein 2 in the crosstalk between endothelial progenitor cells and mesenchymal stem cells. Int J Mol Med 18: 735–739. 16964430 10.3892/ijmm.18.4.735

[49] Kanczler JM, Ginty PJ, White L, Clarke NM, Howdle SM, et al (2010) The effect of the delivery of vascular endothelial growth factor and bone morphogenic protein-2 to osteoprogenitor cell populations on bone formation. Biomaterials 31: 1242–1250. 19926128 10.1016/j.biomaterials.2009.10.059

[50] Kempen DH, Lu L, Heijink A, Hefferan TE, Creemers LB, et al (2009) Effect of local sequential VEGF and BMP-2 delivery on ectopic and orthotopic bone regeneration. Biomaterials 30: 2816–2825. 19232714 10.1016/j.biomaterials.2009.01.031

[51] Wenk E, Merkle HP, Meinel L (2011) Silk fibroin as a vehicle for drug delivery applications. J Control Release 150: 128–141. 21059377 10.1016/j.jconrel.2010.11.007

[52] Villa C, Erratico S, Razini P, Farini A, Meregalli M, et al (2011) In vivo tracking of stem cell by nanotechnologies: future prospects for mouse to human translation. Tissue Eng Part B Rev 17: 1–11. 20846051 10.1089/ten.TEB.2010.0362

[53] Brazelton TR, Blau HM (2005) Optimizing Techniques for Tracking Transplanted Stem Cells In Vivo. Stem Cells 23: 1251–1265. 16109764 10.1634/stemcells.2005-0149

[54] Dupont KM, Sharma K, Stevens HY, Boerckel JD, García AJ, et al (2010) Human stem cell delivery for treatment of large segmental bone defects. Proc Natl Acad Sci U S A 107: 3305–3310. 20133731 10.1073/pnas.0905444107 PMC2840521

[55] Jing XH, Yang L, Duan XJ, Xie B, Chen W, et al (2008) In vivo MR imaging tracking of magnetic iron oxide nanoparticle labeled, engineered, autologous bone marrow mesenchymal stem cells following intra-articular injection. Joint Bone Spine 75: 432–438. 18448377 10.1016/j.jbspin.2007.09.013

