# Supplementary figures and images for: Porous Silk Scaffolds for Delivery of Growth Factors and Stem Cells to Enhance Bone Regeneration
Source: PLoS One. 2014 Jul 22;9(7):e102371. doi: 10.1371/journal.pone.0102371 (PMC4106788; doi:10.1371/journal.pone.0102371)

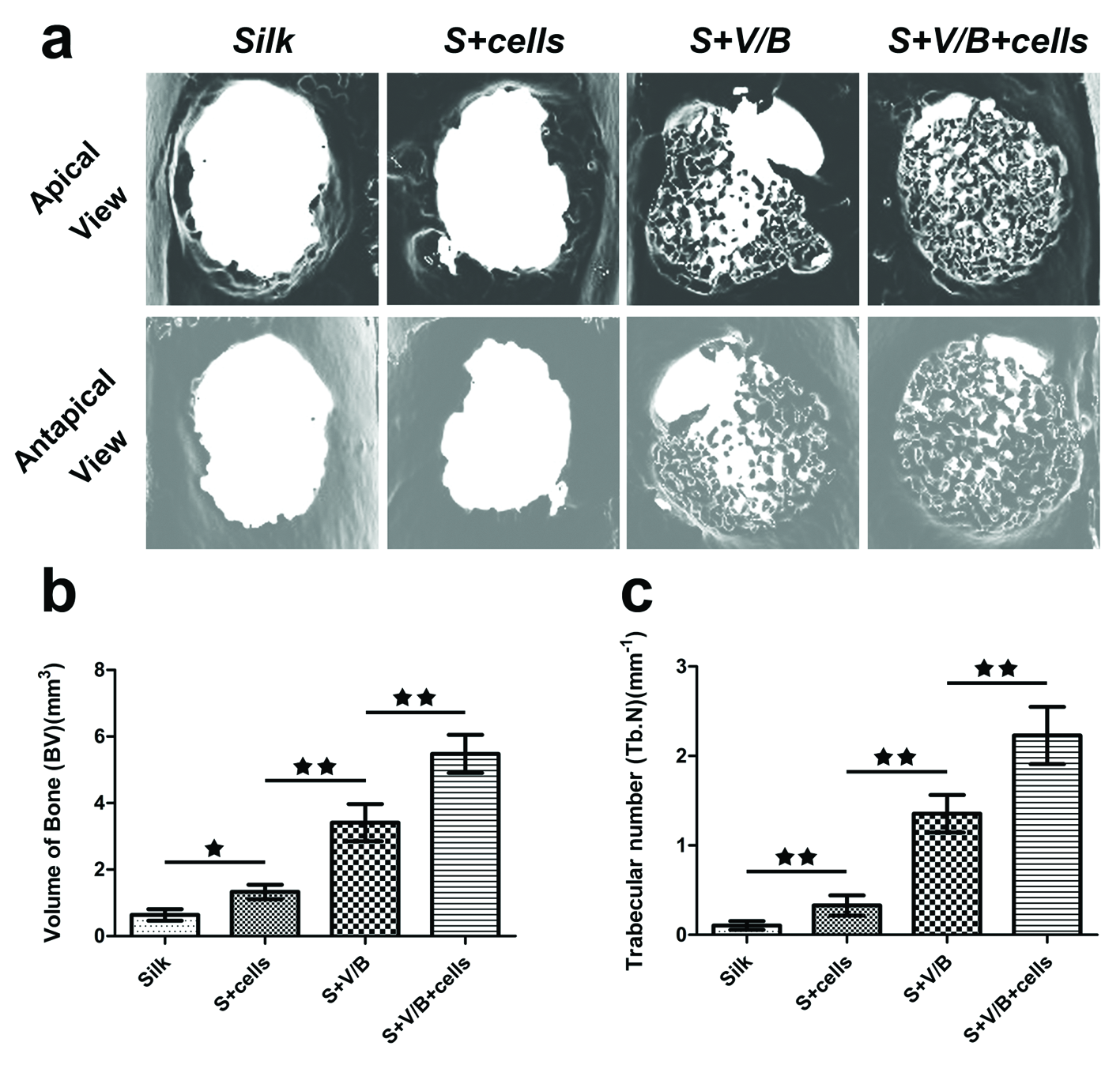

Supplement: Figure S1 — Micro-CT analysis of the repaired skull 8 weeks after implantation. (a) The apical and antapical views of three-dimensional reconstruction image. Bone volume (b) and trabecular number (c) were measured. (★, represents p<0.05; ★★, represents p<0.01). (TIF) [file pone.0102371.s001.tif]

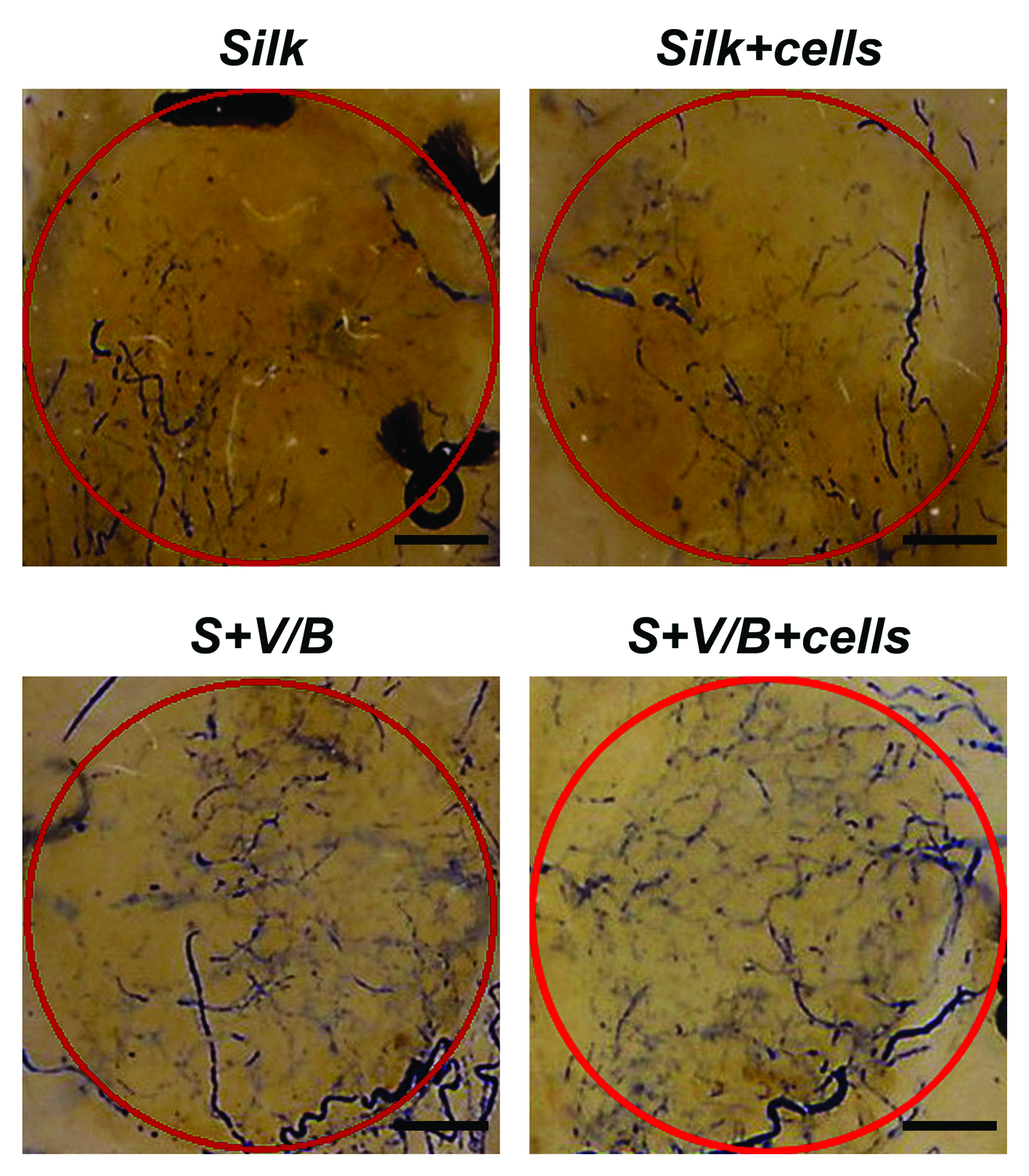

Supplement: Figure S2 — Observation of blue Microfil-perfused blood vessels in the gross specimens. Scale bars are 1 mm. (TIF) [file pone.0102371.s002.tif]

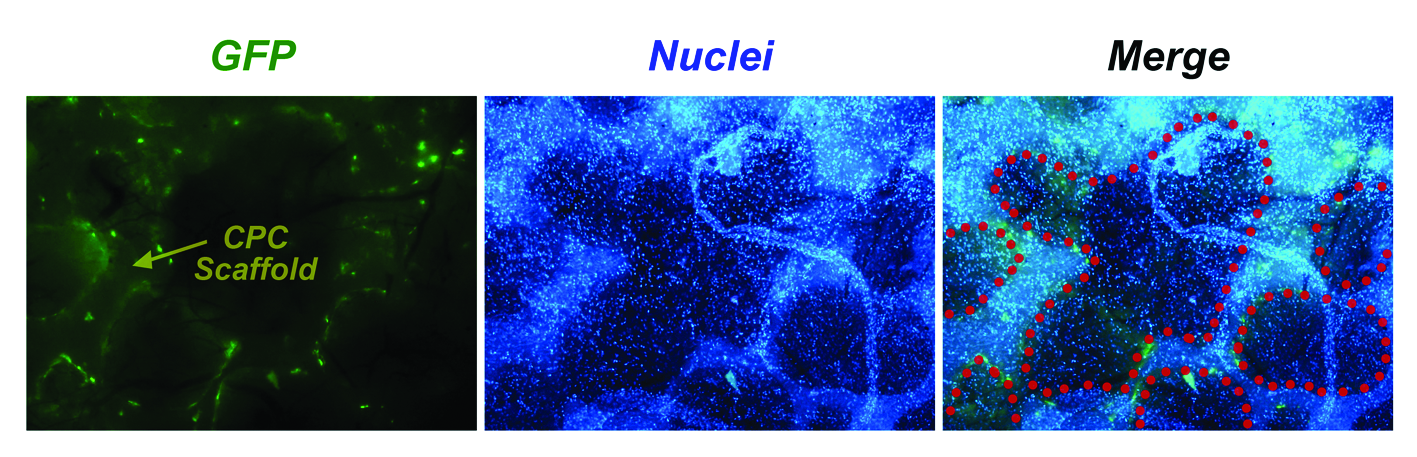

Supplement: Figure S3 — Cell tracking within calcium phosphate cement (CPC) scaffolds. The red dash lines indicates the CPC scaffold surface. (TIF) [file pone.0102371.s003.tif]
